# Supplementary material for: Detailed molecular and epigenetic characterization of the pig IPEC-J2 and chicken SL-29 cell lines
Source: iScience. 2023 Feb 20;26(3):106252. doi: 10.1016/j.isci.2023.106252 (PMC10018572; doi:10.1016/j.isci.2023.106252)
Supplement: Data S1. Complete homer output for identified motifs in Pig IPECJ-2, related to Table 2 — Homer motif analysis results for histone modifications H3K4me1, H3K4me3, H3K27ac, and enhancer elements of pig IPECJ2 cell line. P-values >1e-10 are possible false positives. Within each folder (e.g. peak_files_CTCF) are the html files showing the identified motifs when using homer (e.g. homerResults.html). [file mmc2.zip › S5/Pig_IPECJ_2/peak_files_H3K4me1/homerResults.html]

peak\_files\_H3K4me1/ - Homer de novo Motif Results


# Homer *de novo* Motif Results (peak\_files\_H3K4me1/)

Known Motif Enrichment Results  
Gene Ontology Enrichment Results  
If Homer is having trouble matching a motif to a known motif, try copy/pasting the matrix file into
STAMP  
More information on motif finding results: HOMER
| Description of Results
| Tips
  
Total target sequences = 100425  
Total background sequences = 100502  
\* - possible false positive  

|  |  |  |  |  |  |  |  |  |
| --- | --- | --- | --- | --- | --- | --- | --- | --- |
| Rank | Motif | P-value | log P-pvalue | % of Targets | % of Background | STD(Bg STD) | Best Match/Details | Motif File |
| 1 | T C G A A G C T C A T G G C T A A T C G G C A T G T A C C T G A A G C T A T G C | 1e-305 | -7.036e+02 | 8.24% | 5.38% | 91.1bp (87.3bp) | Fos(bZIP)/TSC-Fos-ChIP-Seq(GSE110950)/Homer(0.986) More Information \| Similar Motifs Found | motif file (matrix) |
| 2 | T A G C C T G A T G C A G C T A G C A T A C G T T G A C G A T C C G A T A C T G | 1e-188 | -4.341e+02 | 20.48% | 16.92% | 86.2bp (86.8bp) | TEAD(TEA)/Fibroblast-PU.1-ChIP-Seq(Unpublished)/Homer(0.913) More Information \| Similar Motifs Found | motif file (matrix) |
| 3 | G A T C A T C G A G T C A C T G A C G T C G T A T C G A G C T A | 1e-120 | -2.781e+02 | 50.85% | 47.16% | 86.1bp (87.4bp) | HOXC13/MA0907.1/Jaspar(0.719) More Information \| Similar Motifs Found | motif file (matrix) |
| 4 | T C G A T G C A C T G A C A G T T C A G G A C T A C G T G C A T T C A G T A G C | 1e-111 | -2.572e+02 | 42.88% | 39.39% | 85.8bp (86.7bp) | Sox5/MA0087.1/Jaspar(0.691) More Information \| Similar Motifs Found | motif file (matrix) |
| 5 | A G T C C G T A C T G A T G C A T C A G A G C T C T A G A T G C | 1e-110 | -2.548e+02 | 54.99% | 51.46% | 85.0bp (88.1bp) | ISL2/MA0914.1/Jaspar(0.799) More Information \| Similar Motifs Found | motif file (matrix) |
| 6 | T G C A G T C A C T G A T G C A T A G C A G T C T A C G A C T G A C G T G A C T | 1e-107 | -2.483e+02 | 47.16% | 43.70% | 85.2bp (87.0bp) | GRHL1/MA0647.1/Jaspar(0.954) More Information \| Similar Motifs Found | motif file (matrix) |
| 7 | A T C G A G T C T C G A A T C G A C G T C A G T G C T A T C G A G T C A G A C T | 1e-102 | -2.366e+02 | 58.60% | 55.22% | 85.0bp (87.9bp) | Unknown(Homeobox)/Limb-p300-ChIP-Seq/Homer(0.746) More Information \| Similar Motifs Found | motif file (matrix) |
| 8 | T A G C T A G C G A T C G A C T C G A T G A T C G A T C T C G A T C A G C T A G | 1e-94 | -2.179e+02 | 48.98% | 45.72% | 84.1bp (88.4bp) | ZBTB7A/MA0750.2/Jaspar(0.757) More Information \| Similar Motifs Found | motif file (matrix) |
| 9 | G T A C C T G A A G T C C T A G G T A C A G T C A G T C C G T A | 1e-94 | -2.169e+02 | 10.61% | 8.72% | 85.6bp (85.5bp) | KLF6/MA1517.1/Jaspar(0.927) More Information \| Similar Motifs Found | motif file (matrix) |
| 10 | C A G T A C T G A G T C A G T C C G A T T C A G T A C G A G T C T G C A T G A C | 1e-87 | -2.025e+02 | 40.60% | 37.53% | 82.8bp (87.5bp) | Tbox:Smad(T-box,MAD)/ESCd5-Smad2\_3-ChIP-Seq(GSE29422)/Homer(0.748) More Information \| Similar Motifs Found | motif file (matrix) |
| 11 | A C G T A C G T C G T A A C G T A G T C C G T A T G A C A T G C | 1e-84 | -1.940e+02 | 33.89% | 31.02% | 85.9bp (87.9bp) | Gata4(Zf)/Heart-Gata4-ChIP-Seq(GSE35151)/Homer(0.854) More Information \| Similar Motifs Found | motif file (matrix) |
| 12 | A T G C G A C T G A C T T C A G C T G A T A G C G T A C C G A T | 1e-83 | -1.928e+02 | 51.55% | 48.48% | 84.6bp (88.1bp) | RAR:RXR(NR),DR0/ES-RAR-ChIP-Seq(GSE56893)/Homer(0.860) More Information \| Similar Motifs Found | motif file (matrix) |
| 13 | A T G C C T G A A T C G A G C T C G T A C G T A C G T A A G C T T C A G T G A C | 1e-82 | -1.894e+02 | 24.75% | 22.20% | 85.8bp (87.1bp) | EWS:ERG-fusion(ETS)/CADO\_ES1-EWS:ERG-ChIP-Seq(SRA014231)/Homer(0.768) More Information \| Similar Motifs Found | motif file (matrix) |
| 14 | C T G A A G T C G A T C A G C T C G T A C T A G A G T C G A C T | 1e-80 | -1.856e+02 | 31.73% | 28.97% | 84.4bp (88.6bp) | Smad4/MA1153.1/Jaspar(0.608) More Information \| Similar Motifs Found | motif file (matrix) |
| 15 | C G A T C A T G G A C T A T G C C G T A T G A C C T G A G T A C | 1e-70 | -1.624e+02 | 34.00% | 31.37% | 85.8bp (86.8bp) | Tbx21(T-box)/GM12878-TBX21-ChIP-Seq(Encode)/Homer(0.796) More Information \| Similar Motifs Found | motif file (matrix) |
| 16 | C G A T T A C G T G A C A C T G A G C T C T G A C T A G C A G T C G A T G C T A | 1e-64 | -1.490e+02 | 10.43% | 8.87% | 84.9bp (87.2bp) | HMBOX1/MA0895.1/Jaspar(0.705) More Information \| Similar Motifs Found | motif file (matrix) |
| 17 | T C A G T A G C G A T C C G A T A G T C C A T G C T A G A G C T C A G T A G C T | 1e-60 | -1.399e+02 | 23.11% | 20.96% | 84.1bp (87.0bp) | IRF5/MA1420.1/Jaspar(0.676) More Information \| Similar Motifs Found | motif file (matrix) |
| 18 | G A T C G T C A G T A C C G T A G T C A G C T A C A T G T A G C A G C T A G C T | 1e-52 | -1.199e+02 | 15.40% | 13.71% | 85.6bp (87.4bp) | Nr2e3/MA0164.1/Jaspar(0.759) More Information \| Similar Motifs Found | motif file (matrix) |
| 19 | A C G T G A C T C A G T G A T C G C T A C T A G T C G A C G T A | 1e-47 | -1.087e+02 | 46.71% | 44.44% | 87.2bp (88.2bp) | ZBTB26/MA1579.1/Jaspar(0.710) More Information \| Similar Motifs Found | motif file (matrix) |
| 20 | T G C A G A T C A G C T G C T A T C G A T A C G A C T G G A T C | 1e-43 | -1.009e+02 | 23.21% | 21.39% | 84.7bp (89.9bp) | TFAP2B(var.2)/MA0812.1/Jaspar(0.665) More Information \| Similar Motifs Found | motif file (matrix) |
| 21 | C A T G A T C G A C T G A G T C A C T G T A G C G T A C G A T C T G C A G T C A | 1e-42 | -9.757e+01 | 19.94% | 18.25% | 82.5bp (87.0bp) | PB0052.1\_Plagl1\_1/Jaspar(0.799) More Information \| Similar Motifs Found | motif file (matrix) |
| 22 | C A G T T C A G G T C A G T C A G A C T A C T G G T C A C T G A C A G T A C T G | 1e-41 | -9.473e+01 | 3.77% | 3.01% | 85.7bp (86.4bp) | PB0028.1\_Hbp1\_1/Jaspar(0.815) More Information \| Similar Motifs Found | motif file (matrix) |
| 23 | G C T A T G A C C A T G C G T A C A G T G A C T C T G A A C T G G A T C G C A T | 1e-36 | -8.359e+01 | 6.32% | 5.39% | 85.7bp (87.4bp) | VENTX/MA0724.1/Jaspar(0.740) More Information \| Similar Motifs Found | motif file (matrix) |
| 24 | A G T C A T G C G A T C A T C G A T C G T A C G A G T C A C T G | 1e-30 | -6.942e+01 | 6.88% | 6.00% | 84.8bp (84.3bp) | PB0103.1\_Zic3\_1/Jaspar(0.721) More Information \| Similar Motifs Found | motif file (matrix) |
| 25 | A T C G A T G C A T C G T A G C A T C G T A C G A T G C A T C G A T G C T A G C | 1e-25 | -5.857e+01 | 2.64% | 2.14% | 85.5bp (82.2bp) | PB0113.1\_E2F3\_2/Jaspar(0.718) More Information \| Similar Motifs Found | motif file (matrix) |
| 26 | A G T C T G C A A G T C A C T G C G A T G A C T C A G T G A C T G T A C C T G A | 1e-25 | -5.789e+01 | 0.26% | 0.12% | 82.0bp (80.3bp) | HIF2a(bHLH)/785\_O-HIF2a-ChIP-Seq(GSE34871)/Homer(0.641) More Information \| Similar Motifs Found | motif file (matrix) |
| 27 | A T G C C G T A G T A C A G T C A C T G G T C A C G A T A G C T | 1e-22 | -5.134e+01 | 1.12% | 0.83% | 81.2bp (84.8bp) | VENTX/MA0724.1/Jaspar(0.745) More Information \| Similar Motifs Found | motif file (matrix) |
| 28 | A G T C T C A G C T A G T C A G A C T G A G C T G A C T T A G C G T C A C G T A | 1e-16 | -3.780e+01 | 0.03% | 0.00% | 82.1bp (0.0bp) | VDR/MA0693.2/Jaspar(0.671) More Information \| Similar Motifs Found | motif file (matrix) |
